# Machine Learning to Detect Alzheimer’s Disease from Circulating Non-coding RNAs

**DOI:** 10.1016/j.gpb.2019.09.004

**Published:** 2019-12-04

**Authors:** Nicole Ludwig, Tobias Fehlmann, Fabian Kern, Manfred Gogol, Walter Maetzler, Stephanie Deutscher, Simone Gurlit, Claudia Schulte, Anna-Katharina von Thaler, Christian Deuschle, Florian Metzger, Daniela Berg, Ulrike Suenkel, Verena Keller, Christina Backes, Hans-Peter Lenhof, Eckart Meese, Andreas Keller

**Affiliations:** 1 Department of Human Genetics, Saarland University, 66421 Homburg/Saar, Germany; 2 Chair for Clinical Bioinformatics, Saarland University, 66123 Saarbrücken, Germany; 3 Institut für Gerontologie, Universität Heidelberg, 69047 Heidelberg, Germany; 4 Department of Neurology, Christian-Albrechts-University of Kiel, 24118 Kiel, Germany; 5 Center for Neurology and Hertie Institute for Clinical Brain Research, Department of Neurodegeneration, University of Tuebingen, 72074 Tuebingen, Germany; 6 German Center for Neurodegenerative Diseases (DZNE), 72076 Tuebingen, Germany; 7 Department of Anesthesiology and Intensive Care, St. Franziskus Hospital Muenster, 48145 Muenster, Germany; 8 Department of Psychiatry and Psychotherapy, University Hospital Tuebingen, 72016 Tuebingen, Germany; 9 Department of Medicine II, Saarland University Medical Center, 66421 Homburg/Saar, Germany; 10 Center for Bioinformatics, Saarland Informatics Campus, 66123 Saarbrücken, Germany

**Keywords:** miRNAs, Neurodegeneration, Alzheimer’s disease, Biomarker, Non-coding RNAs, Gene regulation

## Abstract

Blood-borne small non-coding (sncRNAs) are among the prominent candidates for blood-based diagnostic tests. Often, high-throughput approaches are applied to discover **biomarker** signatures. These have to be validated in larger cohorts and evaluated by adequate statistical learning approaches. Previously, we published high-throughput sequencing based microRNA (miRNA) signatures in **Alzheimer’s disease** (AD) patients in the United States (US) and Germany. Here, we determined abundance levels of 21 known circulating **miRNAs** in 465 individuals encompassing AD patients and controls by RT-qPCR. We computed models to assess the relation between miRNA expression and phenotypes, gender, age, or disease severity (Mini-Mental State Examination; MMSE). Of the 21 miRNAs, expression levels of 20 miRNAs were consistently de-regulated in the US and German cohorts. 18 miRNAs were significantly correlated with **neurodegeneration** (Benjamini-Hochberg adjusted *P* < 0.05) with highest significance for miR-532-5p (Benjamini-Hochberg adjusted *P* = 4.8 × 10^−30^). Machine learning models reached an area under the curve (AUC) value of 87.6% in differentiating AD patients from controls. Further, ten miRNAs were significantly correlated with MMSE, in particular miR-26a/26b-5p (adjusted *P* = 0.0002). Interestingly, the miRNAs with lower abundance in AD were enriched in monocytes and T-helper cells, while those up-regulated in AD were enriched in serum, exosomes, cytotoxic t-cells, and B-cells. Our study represents the next important step in translational research for a miRNA-based AD test.

## Introduction

Alzheimer’s disease (AD) represents one of the most demanding challenges in healthcare [1], [2]. In light of demographic changes and failures in drug development [3], early detection of the disease offers itself as one of the most promising approaches to improve patients’ outcome in the mid- to long term. Especially minimally invasive molecular markers seem to have a significant potential to facilitate a diagnosis of AD, even in early stages.

The importance of minimally invasive molecular markers for AD is reflected by over 3000 original articles and reviews related to AD diagnosis from blood, serum, or plasma samples published and indexed in PubMed. Among the promising approaches are plasma proteomic markers measured by mass spectrometry [4], metabolic patterns [5], gene expression profiles [6], DNA methylation [7], and small non-coding RNAs (sncRNAs) [8]. However, cohort sizes of such studies are often limited and larger validation cohorts frequently did not always match the original results [9]. One of the major challenges is the complexity of signatures that is often required to reach high specificity and sensitivity.

For AD, many miRNA-related studies from tissue [10], blood [11], serum [12], exosomes, [13] or cerebrospinal fluid (CSF) [12] have been performed. In one of the most comprehensive reviews [14], Hu and co-workers investigated 236 papers and reviewed the de-regulated miRNA abundance in different parts of AD patients. In another comprehensive recent review, Nagaraj and co-workers show that out of 137 miRNAs found to exhibit altered expression in AD blood, 36 have been replicated in at least one independent study. Moreover, out of 166 miRNAs being differentially abundant in AD CSF, 13 have been repeatedly found [15].

In previous studies, we performed deep sequencing to measure blood-borne AD miRNA signatures in a cohort of 54 AD patients and 22 controls from the United States (USA) that have been partially validated on a larger cohort of 202 samples by RT-qPCR [8]. In a second study using the same technique, we aimed to validate the results in a patient cohort collected in Germany (GER) that included 49 AD cases, 55 controls and 110 disease controls [16]. The results of both studies were largely consistent with a correlation between both studies of 0.93 (95% confidence interval 0.89–0.96; *P* < 10^−16^).

**Figure 1:**
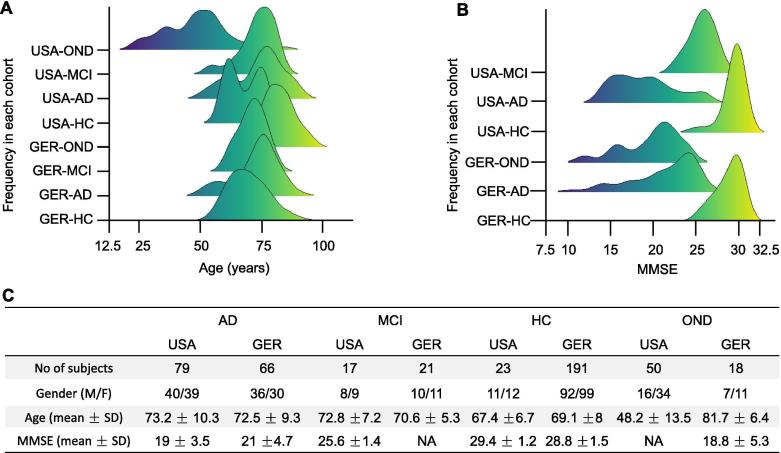
**Distribution of age, gender, diseases, and MMSE** **A.** Histogram for the age distribution in the different cohorts. The diagram shows for each cohort/disease the age distribution. Only the OND group from the US shows a deviation towards younger patients, while all other groups have similar age ranges. **B.** Histogram for the MMSE values. HCs and MCI patients show significantly larger MMSE values as compared to AD and OND patients. **C.** Metrics. For each of the cohorts and diseases, the number of patients in the US and Germany, the mean and SD for age and MMSE as well as the gender distribution are provided. GER, Germany; MMSE, Mini-Mental State Examination; AD, Alzheimer’s disease; OND, other neurological diseases; HC, healthy control; MCI, mild cognitive impairment.

Although deep-sequencing applications are increasingly introduced into clinical care, they are mostly performed for the analysis of DNA or RNAs coding for genes. Small non-coding RNA profiling, however, is mostly achieved by microarray and RT-qPCR based approaches. In the present study, we provide further evidence that blood-borne miRNA signatures can be measured by standard RT-qPCR, becoming valuable tools for the minimally-invasive detection of AD. From our above-mentioned studies and the literature, we selected a set of 21 miRNAs and determined the abundance of these miRNAs in the blood of 465 individuals. The 465 individuals consist of 169 individuals from our initial study (36%) [8], 107 individuals from the second study (23%) [16] as well as 189 newly collected individuals (41%). An overview and summary on the German and US samples is provided in Figure 1A–C, the full details for each individual samples, including age gender, diagnosis, Mini-Mental State Examination (MMSE), and the miRNA measurements, are provided in Table S1.

With the present study we pursue the five main goals to demonstrate that (1) miRNAs from NGS studies can be well reproduced by RT-qPCR experiments; (2) given a reasonable heterogeneity in samples still reproducible measurements in larger cohorts are possible; (3) miRNAs are also correlated to clinical features such as the MMSE value; (4) statistical learning approaches with as few as possible features lead to accurate diagnostic results; (5) the miRNAs likely have functionality in AD via targeting genes.

## Results

### Two endogenous control RNAs show concordant results

Because the selection of the most appropriate endogenous control RNAs for RT-qPCR experiments can be challenging, we previously evaluated systematically whether different endogenous controls lead to differences in miRNA measurements [17]. Especially, most miRNAs seem to be affected by development stages, tissues [18], or diseases [19], limiting their ability as controls and calling for endogenous controls other than miRNAs. Our results suggested that differences can be observed that are however moderate. In the present study we nonetheless evaluated and compared the performance of two commonly used endogenous controls RNU48 and RNU6. Both endogenous controls have been measured in duplicates. In comparing the results, we verified the generally high concordance between the two endogenous controls with a Pearson correlation of 0.854 (95% CI: 0.828–0.877; *P* < 10^−16^). We thus report the result in the current study based on our standard endogenous control RNU48.

In the same direction we also investigated the general stability of RT-qPCR based miRNA measurement. One control sample has been measured 12 times over the study for all miRNAs. The median Pearson correlation coefficient (PCC) exceeded 0.99 as the heatmap and the box plot in Figure S1 show.

### miRNAs are highly significantly correlated with neurodegeneration

**Figure 2:**
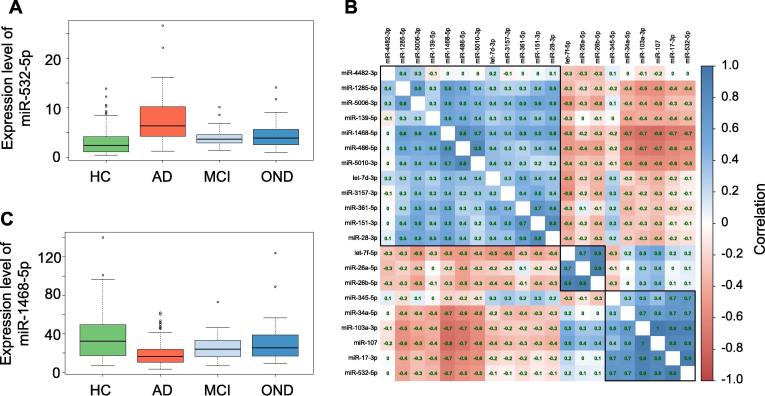
**miRNAs are specifically dysregulated in the four cohorts and are partially co-expressed** **A.** Expression of miR-532-3p. The boxes display the 2nd and 3rd quartile of expression values for miR-532-3p in HC, patients with AD, MCI, or OND. The range of expression values in the four groups is indicated by the error bars with outliers represented by unfilled dots. Median expression of miR-532-3p is indicated as thick black line. **B.** Correlation of miRNA expression. This correlation matrix graphically represents the pair-wise correlation coefficient for all miRNAs tested. According to the color scale on the right side of the matrix, positive and negative correlations are indicated in shades of blue and red, respectively. PCC is given for each pair-wise correlation. Three clusters of miRNAs with highly similar expression patterns are indicated as Clusters A, B, and C on the left side. **C.** Expression of miR-1468-5p. The boxes display the 2nd and 3rd quartile of expression values for miR-1468-5p in HC, patients with AD, MCI, or OND. The range of expression values in the four groups is indicated by the error bars with outliers represented by unfilled dots. Median expression of miR-1468-5p is indicated as thick black line. PCC, Pearson correlation coefficient.

In total, 465 participants have been analyzed by RT-qPCR. The abundance levels of 18 of the 21 miRNAs were significantly different between the four groups considered, *i.e.*, AD, mild cognitive impairment (MCI), other neurological diseases (OND), and healthy controls (HC). With an Benjamini-Hochberg (BH) adjusted *P* value of 4.8 × 10^−30^, the most significant miRNA was miR-532-5p, which showed markedly decreased levels in AD patients, and slightly decreased levels in patients with OND and MCI (Figure 2A). The abundance levels of miR-17-3p, the miRNA with the second lowest *P* value (*P* = 8.8 × 10^−28^), showed a similar pattern as miR-532-5p (PCC > 0.9). The overall correlation matrix between the 21 miRNAs showed three large clusters of miRNAs with similar expression in the following referred to as Clusters A, B, and C (Figure 2B). The third and fourth most significant miRNAs in ANOVA, *i.e.*, miR-103a-3p and miR-107 (*P* = 2.4 × 10^−18^ and *P* = 3.6 × 10^−15^, respectively), came from Cluster C, like miR-532-5p, and miR-17-3p. MiR-1468-5p (Cluster A, *P* = 6.2 × 10^−12^; Figure 2C) shows an opposite expression pattern, i.e. a higher abundance in AD patients as compared to HC. The boxplots in Figure 2A/2C also underline that the deregulation of these miRNAs is strongest in AD compared to the HC. There is, however, a deregulation in MCI or OND, but to a lesser extent, such that the altered abundance is at least partially specific for AD. This result is consistent with our previous work based on high-throughput sequencing.

For a more detailed understanding of the miRNAs and their correlation to AD and other factors, we next assessed whether the abundance levels were correlated to age or gender, or, in case of AD and MCI with the MMSE results (Table 1). As Table 1 highlights, none of the miRNAs was associated with gender and five miRNAs were weakly associated with age of patients. Following adjustment for multiple testing, 14 miRNAs showed a significant differential expression in AD patients compared to controls (*i.e.*, HC, MCI, and OND combined). The above mentioned miR-532-5p and miR-17-3p were again the most significant markers for AD. Furthermore, ten miRNAs were significantly correlated with the MMSE value. Interestingly, all three miRNAs of Cluster B (Figure 1B), *i.e.*, miR-26a, 26b-5p, and let-7f-5p, showed the highest significance for the correlation to MMSE (*P* < 0.005). Since neither all miRNAs nor the MMSE values were normally distributed we repeated the analyses with non-parametric and ranked based Spearman correlation coefficient (SCC), overall leading to comparable results (see Table S2).

**Table 1:** **Raw and adjusted *P* values of miRNAs for age, gender, AD, and MMSE**

*Note*: *P* values for gender and AD were calculated based on *t* test; *P* values for age and MMSE were calculated based on Pearson's product moment correlation coefficient. *P* values were adjusted by the Benjamini-Hochberg procedure. Adjusted *P* values <0.05 are indicted in orange and those <0.005 are put in bold with blue background. AD, Alzheimer’s disease; MMSE, Mini-Mental State Examination.

Besides the comparison of healthy controls to AD we also asked whether MCI patients can be separated from AD patients using miRNAs. Indeed, eleven miRNAs had significant differential expression in MCI versus AD following adjustment for multiple testing: miR-17-3p (*P* = 10^−12^; down in AD), miR-532-5p (*P* = 8 × 10^−10^; down in AD), miR-103a-3p (*P* = 10^−8^; down in AD), miR-107 (*P* = 4 × 10^−7^; down in AD), let-7d-3p (*P* = 9 × 10^−7^; up in AD), let-7f-5p (*P* = 3 × 10^−5^; down in AD), miR-345-5p (*P* = 0.0002; down in AD), miR-26a-5p (*P* = 0.002; down in AD), miR-26b-5p (*P* = 0.009; down in AD), miR-1468-5p (*P* = 0.02; up in AD), and miR-139-5p (*P* = 0.03; up in AD).

### miRNA profiles from the US and German cohort show consistent results

**Figure 3:**
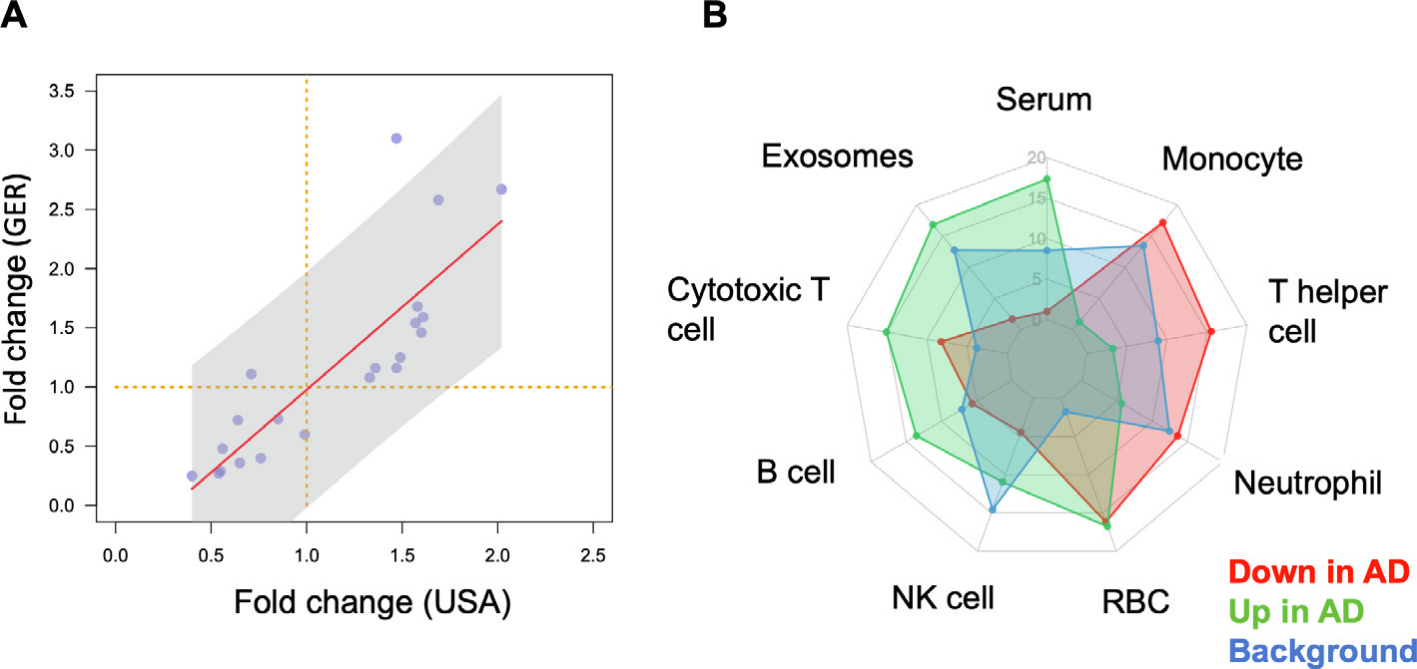
**Differentially-expressed miRNAs are concordantly expressed in the German and the US cohorts and belong to specific blood compounds** **A.** Fold change in the USA cohort compared to the GER cohort. The X- and Y-axes represent the fold change between AD and HC on a log_2_ scale for the USA and GER patient cohorts, respectively. Each miRNA is represented by one dot. The dashed orange line is the segregation between up- and down-regulation. miRNAs in the upper right or lower left quadrant are concordantly up- or downregulated in AD compared to HC in both cohorts, respectively. The solid red line is a linear regression fit and the shaded area is the 95% confidence interval of that fit. **B.** Radar chart showing the blood compound distribution. The plot shows the relative abundance of up-regulated, down-regulated, and all miRNAs in different blood compounds. Since the relative abundance is provided, it is more appropriate to compare the different groups within one specific compound rather than comparing different compounds to each other.

It is essential to understand whether biomarkers can be concordantly determined in different cohorts. Although a direct comparison of ethnic groups was not in the scope of our analysis we nonetheless asked whether miRNA profiles for one disease measured on two different continents are concordant to each other. We thus compared the profiles measured from GER and USA cohorts. As the GER cohort was about twice as large as the USA cohort and *P* values depend on the number of individuals in each cohort, a comparison based only on *P* values is potentially biased. Therefore, we computed the fold changes (on a logarithmic scale) between AD and controls (Figure 3A). In this plot miRNAs in the upper right quadrant are down-regulated and miRNAs in the lower left quadrant are up-regulated in AD compared to controls concordantly in both cohorts. Of 21 miRNAs, only miR-4482-3p was down-regulated in the GER cohort, but up-regulated in the USA cohort. The differences in abundance levels of this miRNA in AD compared to controls were, however, not significant, neither in the GER nor in the USA cohort, nor in the combined analysis. Thus, miR-4482-3p likely represents a single false positive marker from the initial deep-sequencing based miRNA discovery study. In contrast, the results for the remaining 20 miRNAs were concordant between the USA and the GER cohort. Furthermore, eleven of these miRNAs were nominally significant in both cohorts, when analyzing the USA cohort and the GER cohort separately, and remained significant in the combined analysis. These significant miRNAs include miR-103a-3p, miR-107, miR-1285-5p, miR-139-5p, miR-1468-5p, miR-17-3p, miR-28-3p, miR-361-5p, miR-5006-3p, miR-5010-3p, and miR-532-5p.

### Up- and down-regulated miRNAs are expressed in different blood compounds

We asked whether the miRNAs that are up- and down-regulated are expressed to the same amount in different blood cell types, serum or exosomes. To this end we made use of a public miRNA blood cell type atlas [20]. For the up- and down-regulated miRNAs we then compared the average expression in the different compounds and compared them to the background distribution of all human miRNAs (Figure 3B). Interestingly, we observed a highly specific pattern. miRNAs up-regulated in AD were expressed mostly in serum, exosomes, cytotoxic t-cells, and b-cells while those that were down-regulated in AD were expressed in monocytes and t-helper cells. These results suggest a complex regulatory pattern of miRNAs in the different blood cell compounds which would have been likely not observed if only a specific blood cell type or serum would have been investigated.

### Machine learning facilitates accurate diagnosis of AD

**Figure 4:**
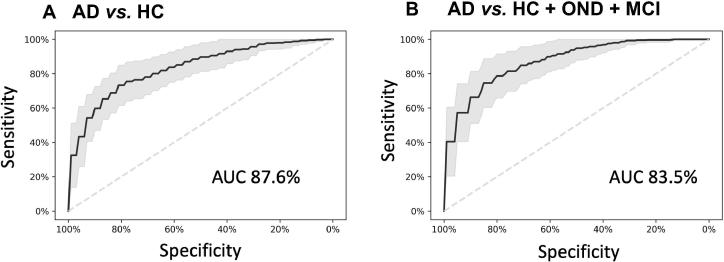
**miRNA classifiers show a high diagnostic performance to detect AD** Diagnostic performance of the miRNA classifiers. **A.** ROC AUC for the diagnosis of AD patients compared to HC. **B.** ROC AUC for the diagnosis of AD patients compared to all controls combined (HC, MCI, and OND). The black line indicates the average ROC values of all replicates and folds of the 5 × 10-fold cross-validation models, and the gray area represents the resulting standard deviation. The average AUC obtained over all replicates and folds is displayed for each classification scenario. ROC, receiver operator characteristics; AUC, area under the curve.

To obtain more accurate diagnostic results, molecular markers can be considered as “weak learners” that can be combined by machine learning approaches. For our present data set, we explored common statistical and deep learning approaches including support vector machines, decision trees, neural networks and gradient boosted trees and others using five repeated runs of a ten-fold cross validation. While the performance of all approaches was similar (data not shown), the best results were obtained by gradient boosted trees. Compared to other classifiers, gradient boosted trees have the additional advantage that missing values do not have to be imputed. In the classification, two scenarios were modeled: First, the diagnosis of AD patients with unaffected controls (HC) as background group, and second, the diagnosis of AD patients with all controls, *i.e.*, HC, OND, and MCI combined, as background group. In the first and apparently less complex scenario the gradient boosted tree model reached an area under the curve (AUC) of 87.6% (Figure 4A). For the second and more complex case, an AUC of 83.5% was reached (Figure 4B). A further advantage of the gradient boosted tree models is that sensitivity and specificity can be well balanced and traded-off. Depending on whether a diagnosis trimmed for sensitivity or for specificity is required *e.g*., in screening tests, as confirmatory tests or tests for enrollment for clinical studies, a sensitive or a specific model can be chosen.

Feature importance values for each miRNA based on the relative gain obtained via their splits were extracted from both models using the method provided by LightGBM (Table S3) According to this metric, miR-17-3p had the highest importance value in both models, followed by miR-5010-3p. For the model comparing AD to all controls, the next most important miRNAs were let-7d-3p, miR-26b-5p, and miR-28-3p. For the model comparing to unaffected controls, miR-361-5p, let-7d-3p, and miR-532-5p were the next most important features. Interestingly, let-7d-3p and miR-26b-5p were not significantly associated with AD on their own, suggesting that their discriminative power might come from the combination with other miRNAs or their association with different stages of the disease. For example, miR-26b-5p was recently reported to be likely deregulated early in AD, even before the appearance of clinical symptoms [21].

### miRNAs are enriched in specific functional categories

**Figure 5:**
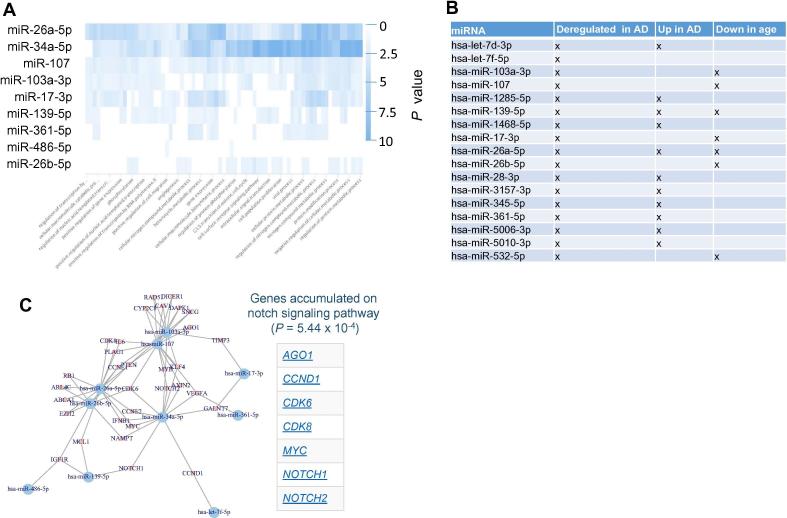
**AD miRNAs regulate distinct pathways and form a dense regulatory core network** **A.** Heatmap of the miRPathDB results. The heatmap presents the negative decade logarithm of miRNAs and target pathways, and the color represents the significance values. **B.** Overview of miRNAs in significant categories. For the three significant miEAA categories we highlight the miRNAs participating in the respective categories. **C.** miRNA target network from miRTargetLink. From miRTargetLink we extracted the target network of the miRNAs and generated a representation in R using the igraph library. Each node is a miRNA/gene and an edge means that the miRNA targets that gene. As an example of an enrichment of target genes, the genes on the Notch pathway are shown on the right side of the network.

To get insights into the targeting of the dysregulated miRNAs, we performed different miRNA target analyses. First, we individually searched for each miRNA those pathways that are enriched with target genes of that miRNA. The result is presented as heat map in Figure 5A. Most significant pathways were computed for miR-34a-5p miR-26a-5p followed by miR-107. Among the pathways, many transcription regulated categories have been observed. This result is however to be expected since the main biological function of miRNAs is to regulate the gene expression.

To get more insights, we next performed a miRNA Enrichment analysis [22]. Following adjustment for multiple testing, we identified three categories to be significantly enriched including “Dys-regulation in AD” (*P* = 4.8 × 10^−8^), “Up-regulation in AD” (*P* = 0.00018), and “Age” (*P* = 0.02). Two of three categories were directly related to AD. Also this is an expected result for miRNAs that were known to be associated with AD. In addition, these miRNAs are negatively correlated with age. Although this was a weak correlation, it still suggests that the abundances of these miRNAs are lower in older patients. Figure 5B presents for each miRNA in the signature on which categories it has been observed. Performing an enrichment analysis for each of the three miRNAs clusters indicated in Figure 2B, we found cluster A to be especially enriched with miRNAs that are “up-regulated in AD” (*P* = 4.9 × 10^−6^) while for cluster B the only significant category was “down-regulated in AD” (*P* = 0.04).

In a third analysis we analyzed all target genes of the miRNAs that had strong evidence in the miRTarBase and were extracted from miRTargetLink. This analysis highlighted that for most miRNAs in our signature, target genes that have been experimentally validated are known. The target network shown in Figure 5C highlighted a dense structure. This network was enriched for genes associated with AD including ABCA1, DAPK1, IGF1R, and VEGFA according to the national institute of aging (NIA). Likewise, “DNA damage response” represented by CCND1, CCNE1, CCNE2, CDK6, MYC, RAD51, and RB1 was over represented. Moreover, the genes in that network were also enriched for the notch signaling pathway.

## Discussion

In the current study we present results of our ongoing efforts to develop a diagnostic test for AD patients based on circulating miRNA profiles extracted from blood cells.

As Figure 1 and Table S1 highlight, the samples were largely homogenous with respect to the age and gender distribution. With respect to other characteristics the cohort was however heterogenous (*e.g.*, the origin of the samples from two continents, different diagnostic procedures to identify the patients, potentially different treatment regimens, or a spectrum of patients with higher and lower MMSE values). This heterogeneity helps us to understand whether the de-regulation in miRNA patterns between AD patients and controls is of general nature and helps to assess whether *e.g.*, miRNAs are associated with the MMSE state.

The current outcomes are consistent with our previous studies in the US and Germany on smaller cohorts. In contrast to the previous studies relying on deep sequencing, we here applied RT-qPCR as molecular profiling technique that can be more easily driven towards application in clinical care. In the context of the known variability and the bias introduced by sample integrity and sample treatment [23], [24], [25] in deep sequencing data, RT-qPCR offers a promising alternative for routine application. But also for RT-qPCR experiments, there is a debate whether RNA samples with low integrity, *i.e.*, low RIN values, compromise miRNA expression data [26], [27]. In our study, we also measured RIN as quality criterion for RNA integrity of the samples. The markers that we validated in this study seem to play partially an important role in different diseases. As an example, our most significant marker miR-532-5p is not only correlated and functionally associated to cancer [28], [29], [30]. The miRNA and its target network is also associated to sporadic amyotrophic lateral sclerosis [31]. Further, the miR-532-5p has also been discovered in exosomes of multiple sclerosis patients [32] and in exosomes of patients with the geriatric frailty syndrome [33]. Also, our analyses indicate a very essential role of exosome derived miRNAs.

The results of the two cohorts from the US and from Germany were highly concordant. As to be expected by the selection of AD-associated miRNAs for this study, the miRNAs and the target genes of the miRNAs were both significantly associated with the development of AD. Our test that is highly reproducible can be applied with a model based on specificity, sensitivity or trimmed for overall performance. The quality of the results is indicated by an AUC of 87.6% for the comparison between AD and unaffected controls, and an AUC of 83.5% for a comparison between AD and a combined group of unaffected controls, MCI patients and patients with OND. It is known that complex statistical learning approaches can lead to overfitting, especially considering the curse of dimensionality [34] and the fact that usually many more features (*p*) are measured as compared to the number of patients (n), the *p*≫*n* problem. In our study we however measured *p* = 21 markers and *n* = 465 individuals. Further, we even select small subsets of these markers for our models and perform comprehensive re-sampling to prevent potential overfitting. Although the de-regulation of miRNAs was generally concordant between the GER and the USA cohort, miRNAs have shown differences in the expression level in the two cohorts. This might be due to technical reasons such as shipment, other batch effects or biological differences. Despite this fact, the statistical learning approach succeeded to separate AD and controls in the GER and the USA cohort. In sum, the performance of our diagnostic solution compares well to other recently-developed tests, such as the plasma amyloid marker introduced by Nakamura and co-workers [4]. While already such single “omics” tests have a large potential, the targeted combination of few representatives from different “omics” classes can add even more diagnostic information, supporting clinicians in detecting AD patients in time. One challenge of respective studies is that the clinical diagnosis may be imperfect. The MCI patients that are an important second control group besides the unaffected controls may have already early forms of AD that are not yet detected with the current diagnostic means.

A pathway based analysis of miRNAs and target genes indicated a functional role of the miRNAs. This is further supported by a different blood compound distribution of those miRNAs that are up- and down-regulated in AD. Respective pathway analyses have however always considered with caution, especially when small sets of miRNAs are considered. Although the results of the analysis seem to be reasonable, a potential bias is hard to be excluded. *e.g.*, we picked already miRNAs known from literature to be associated with AD. An enrichment of AD related miRNAs itself is thus an expected result. Similarly, also the target gene analyses might be biased for miRNAs and target genes that are in the focus of many research groups.

As for other omics types, confounders including age and gender potentially influence also the results of miRNA biomarker studies [35]. To minimize the influence of such confounders, our cohorts largely show similar age and gender distribution (Table 1). In addition, we investigated the influence of the age and gender on the miRNA profiles. Except for a very modest influence of age, we found no evidence for an influence of these confounders on the miRNA pattern. Notably, miRNAs that are down-regulated in AD were partially expected to be lower expressed with increasing age in a normal population. Among the many different candidates for minimally-invasive and potentially early stage tests for AD, our study indicates that circulating miRNAs likely in combination with other blood-born omics profiles will contribute to stable tests applicable to specific diagnostic questions with regard to this highly complex disease.

## Materials and methods

### Overview of the study

In the current study we included patients from the US [8] and Germany [16] that were partially collected within the longitudinal Tübinger Erhebung von Risikofaktoren zur Erkennung von Neurodegeneration (TREND) study. From the former studies we included those individuals, where a sufficient amount of high-quality RNA was left for analysis. In detail, 169 individuals from our initial study (36%) [8], 107 individuals from the second study (23%) [16], as well as 189 newly collected individuals (41%) were included in the study. The studies were approved by the institutional review boards of Charité – Universitätsmedizin Berlin (EA1/182/10), or the ethical committee of the Medical Faculty of the University of Tuebingen (Nr. 90/2009BO2). All subjects gave written informed consent. Besides AD patients and HC, patients with OND such as Parkinson’s disease (PD), schizophrenia or bipolar disorder were included and grouped together, termed OND. Further, patients with MCI were included to evaluate the specificity of the miRNA markers for AD. For each of the four groups and separately for the USA and GER cohorts, total number, age, gender distribution, and MMSE value are presented in Table 1. Moreover, from one individual, 12 technical replicates were measured continuously during the project as process control.

### miRNA marker set selection

From our two previous studies [8], [16] we selected the top miRNAs that were concordant between the two studies, and also checked for evidence that the miRNAs are associated with AD in literature. A final set of 21 miRNAs was selected. These are listed in Supplemental Table 4 where additional selection criteria are provided. In more detail, 17 miRNAs were significantly associated with AD in our first study, 14 miRNAs were significant in our second study. miR-34a-5p was not detected in our previous studies by NGS but in a study by Cosin-Tomas [36]. Further, this miRNA is one of our main targets regulating calcium signaling, NFKappaB pathway and T-cell killing and is down-regulated significantly in aging [37], [38]. miR-151-3p is one of the most stable miRNAs in our studies as well as miR-486-5p, which is a red blood cell miRNA that serves as positive control [20].

### RNA extraction and quality control

Total RNA from PAX-Gene Blood Tubes (Catalog No. 762165, BD Biosciences, Franklin Lakes, NJ) was isolated using the Qiacube robot with the PAXgene Blood miRNA Kit (Catalog No. 763134, Qiagen, Hilden, Germany) according to manufacturer’s instructions. In the tubes, 2.5 ml blood are collected, typically yielding around 1 mg total RNA. RNA quantity and quality were assessed using Nanodrop (Thermo Fisher Scientific) and RNA Nano 6000 Bioanalyzer Kit (Catalog No. 5067-1511, Agilent Technologies, Santa Clara, CA). Mean RNA integrity number (RIN) value of the RNA samples was 7.5 (STDEV 1.4).

### RT-qPCR

Quantification of miRNAs was performed using miScript PCR system and custom miRNA PCR arrays (all reagents from Qiagen, Hilden, Germany). Custom miRNA PCR arrays were designed in 96-well plates to measure the expression of 21 human miRNAs and RNU48 as well as RNU6 as two endogenous controls in duplicates. Two process controls (miR-TC for RT efficiency, PPC for PCR efficiency) were included as single probes. A total of 100 ng total RNA was used as input for reverse transcription reaction using miScriptRT-II kit according to manufacturer’s recommendations in 20 µl total volume (Catalog No. 218161). Subsequently, 1 ng cDNA was used per PCR reaction. PCR reactions with a total volume of 20 µl were setup automatically using the miScript SYBR Green PCR system (Catalog No. 218076) in a Qiagility pipetting robot (Qiagen, Hilden, Germany) according to manufacturer’s instructions. Data from samples that failed the quality criteria for the process controls was excluded, leaving expression data from 465 samples available for analysis. For process control over the course of the project, eleven technical replicates of one cDNA sample were measured throughout the course of the project to estimate technical reproducibility. We computed 55 pair-wise correlation coefficients between any pair of the replicates and found a median correlation of 0.996, indicating high technical reproducibility of our assay.

### Statistical approaches

From the Cq values, delta Cq values in relation to the endogenous control (RNU48) were computed. Mean delta Cq value per individual was scaled to zero. Missing values were not imputed. As estimate of the expression on a linear scale, 2^deltaCq^ values were computed. For multi group comparisons, Analysis of Variance (ANOVA) was performed. Since the miRNA data and partially the response variable were not always normally distributed according to Shapiro Wilk tests, we performed for the pair-wise comparisons and for the correlation analysis parametric as well as non-parametric tests. For pair-wise comparisons, both, parametric t-test and non-parametric Wilcoxon Mann-Whitney test were calculated. If not mentioned explicitly and where applicable, all *P* values were adjusted for multiple testing by the Benjamini-Hochberg approach. For correlating miRNAs to the age and the MMSE value, the *P* value was computed based on parametric Pearson's product moment correlation coefficient as well as non-parametric Spearman Correlation. To find enrichment of miRNAs in specific blood compounds we used data of an NGS based blood cell miRNA repertoire [20]. Each miRNA was normalized to 100% and the different expression ratios in the different blood compounds were compared to each other.

### miRNA target analysis

We performed three different approaches on miRNA target analysis. First, for each single miRNA the target pathways have been extracted from miRPathDB [39] and the CustomHeatmap tool was used to find miRNAs that target at least 5 pathways and pathways targeted by at least 5 miRNAs from biological GO processes. Next, we performed a so-called miRNA set enrichment analysis relying on the hypergeometric distribution using MIEAA [22]. Here, the miRNAs in the study were compared to the background distribution of all miRNAs and the procedure was repeated for the dys-regulated miRNAs. All pathways with an adjusted *P* value below 0.05 were considered to be significant. Finally, we used the miRTargetLink tool [40] to extract the experimentally validated targets of the miRNAs. In this analysis only the strong target category from miRTarBase has been used to obtain specific results. From that data we computed a network using the R igraph package and performed an enrichment analysis of the target genes in that network.

### Machine learning

A prediction model based on the RT-qPCR Cq values was developed using gradient boosted trees from the LightGBM framework (version 2.1.0). Since not all miRNAs were consistently measured for all patients, tree-based methods are particularly suited for this task, as they can handle missing values and no imputation is required. LightGBM ignores the missing values when computing the splits of the trees and assigns all samples with missing values to the side that reduces the loss most. The performance of the model was assessed using five repetitions of stratified ten-fold cross-validation using scikit-learn 0.19.1 with Python 3.6.4 [41]. Each repetition was initiated with an integer seed (0–4). Thus, in total 50 combinations of different training and validation sets were considered. The reported ROC AUC corresponds to the average performance over all repetitions and folds of the model, on data not used for training. The models were manually tuned (*i.e.*, no grid search was performed) over the number of leaves (testing ranges between 5 and 50), number of estimators (between 40 and 120), learning rate (0.01 to 0.2), and depth (3 to no restriction). The final model comparing patients with AD to all controls uses 30 leaves, a learning rate of 0.1 and 100 estimators. The model comparing patients with AD to unaffected controls uses 9 leaves, a learning rate of 0.05 and 100 estimators. The depth of both models was not restricted. Gradient boosted trees outperformed other tree-based methods such as random forests, or classifiers as Support Vector Machines or Neural Networks (data not shown). As an input for the classification task, the expression matrix of the delta Cq values has been used.

## Data availability

The full data set is available as Table S1 without any restrictions.

## Authors’ contributions

NL measured the samples and supported the interpretation of data; TF and FK interpreted and analyzed the data; MG, WM, CS, AKvT, CD, FM, US, and DB supported the study conceptionally, added to the study protocol and enrolled patients; VK added to the clinical interpretation of the data; CB supported the data analysis and contributed to drafting the manuscript; SD and SG supported to measure and interpret the data; HPL, EM, AK were the PIs of the study, contributed to writing and correcting the manuscript and to data analysis and interpretation.

## Competing interests

The authors have declared no competing interests.

## Supplementary material

Supplementary Figure S1 **Heatmap and box plot showing the reproducibility of 12 replicated measurements** The right hand side of the plot shows a box plot for all correlation values in the heat map.
