# Supplementary figures and images for: Machine Learning to Detect Alzheimer’s Disease from Circulating Non-coding RNAs
Source: Genomics Proteomics Bioinformatics. 2019 Dec 4;17(4):430–40. doi: 10.1016/j.gpb.2019.09.004 (PMC6943763; doi:10.1016/j.gpb.2019.09.004)

## Slide 1
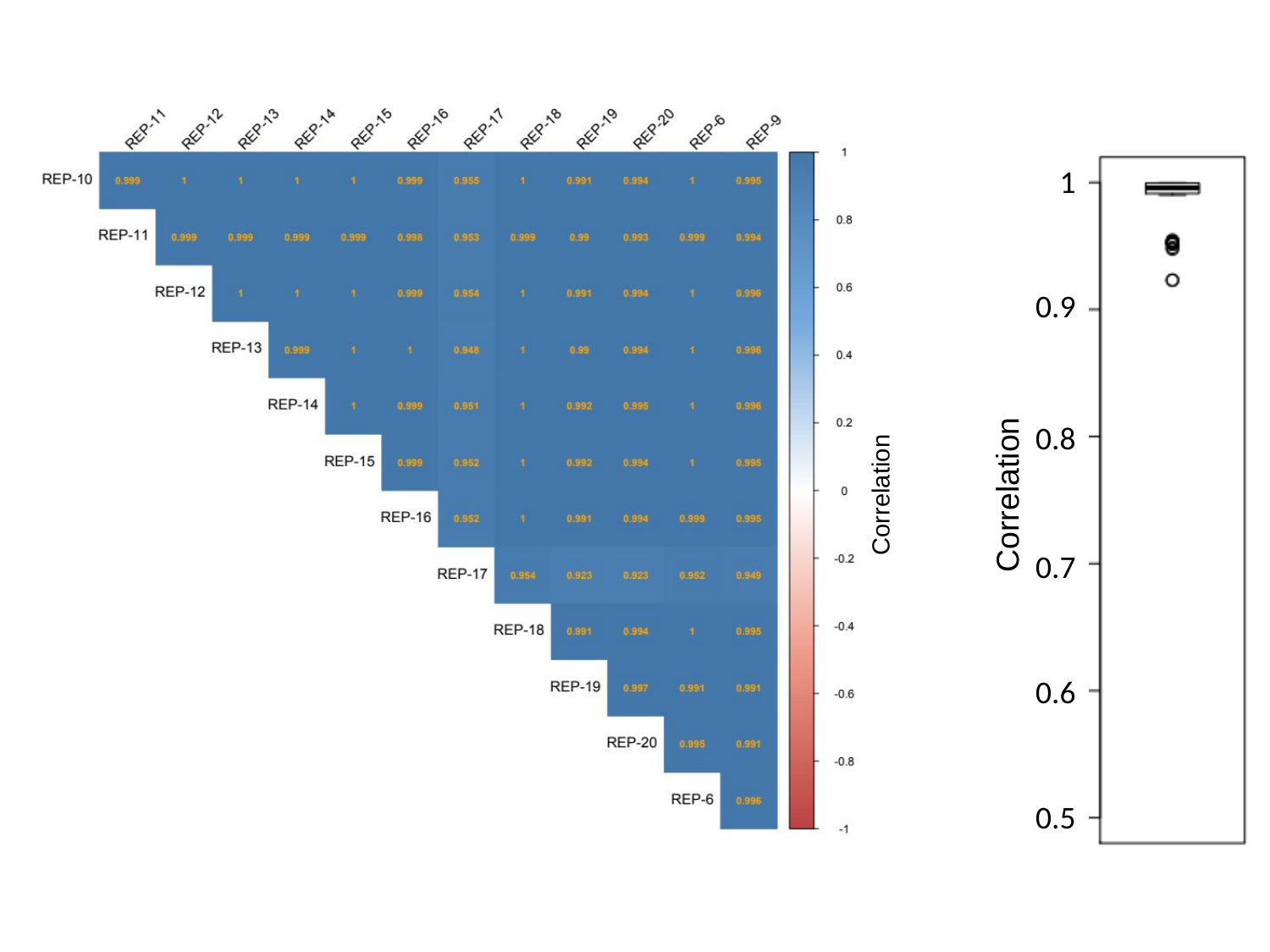

1
0.9
0.8
Correlation
Correlation
0.7
0.6
0.5

Supplement: Supplementary Figure S1 — Heatmap and box plot showing the reproducibility of 12 replicated measurements The right hand side of the plot shows a box plot for all correlation values in the heat map. [file mmc1.pptx]
